# Charting Advances in Asset Management Systems: A Bibliometric Analysis Revealing Applications and Potential in Healthcare

**DOI:** 10.3390/healthcare13222979

**Published:** 2025-11-19

**Authors:** Dalibor Stanimirović, Lan Umek, Dejan Ravšelj

**Affiliations:** Faculty of Public Administration, University of Ljubljana, 1000 Ljubljana, Slovenia; lan.umek@fu.uni-lj.si (L.U.); dejan.ravselj@fu.uni-lj.si (D.R.)

**Keywords:** asset management system, healthcare, medical equipment, bibliometric analysis

## Abstract

**Background:** Asset management has become crucial to organizational performance over the past three decades. Implementing an Asset Management System (AMS) can be pivotal in managing the operation, sustainability, and efficiency of both tangible and intangible organizational assets. However, many organizations still underappreciate AMSs, particularly in healthcare, where poor organization, unclear processes, and a lack of control contribute to long patient waiting times, financial losses, regulatory non-compliance, and diminished credibility. **Methods:** This study provides a comprehensive review of the existing body of research on AMSs, discusses AMSs in the context of healthcare, and identifies the specific healthcare areas that have most frequently been the focus of AMS research. This study applies bibliometric analysis of 16,667 documents on AMSs, complemented by a focused bibliometric analysis of a subset of 248 publications specifically addressing AMSs in healthcare. All documents, published up to the end of 2024 and indexed in the Scopus database, were analyzed to investigate the evolution of AMS research, with a particular emphasis on its applications within healthcare. The research employs several bibliometric approaches, utilizing the Python and VOSviewer software. **Results:** The findings highlight the rapid growth of AMS research, evolving from a niche topic into a strategic discipline that enhances predictive maintenance, efficiency, and sustainability. In healthcare, the adoption of AMSs has grown substantially, supported by the integration of artificial intelligence (AI) and the Internet of Things (IoT). **Conclusions:** The incorporation of these technologies has enabled more effective monitoring of medical equipment, improved oversight of critical infrastructure, and optimized the operational performance of healthcare providers. Nevertheless, significant research gaps remain concerning the direct impact of AMSs on the quality of patient care, provider coordination, and strategic decision-making. Addressing these gaps is essential not only for advancing academic knowledge but also for leveraging the full potential of AMSs to enhance healthcare delivery, improve outcomes, and support the evidence-based management of healthcare systems.

## 1. Introduction

Over the past three decades, the lack of well-established methods and practices for effective asset management has driven organizations to seek more efficient ways to enhance asset performance. Initially, many organizations relied on trial and error to optimize asset utilization, which led to the development of guidelines based on accumulated experience and knowledge [1,2]. These guidelines have since been standardized and disseminated internationally, resulting in asset management becoming a key factor in organizational performance and long-term growth [3]. The concept of asset management has evolved significantly since its inception in the 1970s, when it was primarily associated with the optimized maintenance of physical assets. Initially, asset management focused on balancing financial and operational considerations to ensure cost efficiency throughout an asset’s lifecycle, a practice referred to as economic asset management [4,5]. Over time, advancements in information and communication technologies (ICTs) have further promoted the adoption of asset management and allowed asset management to become more structured and data-driven [6]. The introduction of sophisticated software solutions and emerging technologies such as radio frequency identification (RFID), the Internet of Things (IoT), blockchain, and artificial intelligence (AI) has further transformed the field, enabling improved asset tracking, predictive maintenance, and operational efficiency [7,8,9].

The term “asset” carries various meanings across different sectors but is generally defined as any entity that holds value for an individual, company, or organization. Assets can be categorized into five broad types: financial, human, informational, intangible, and physical. A central principle of asset management is maximizing the value derived from these assets, which requires an effective and integrated management approach. According to widely accepted definitions, asset management encompasses the coordinated activities of an organization to realize value from assets. More precisely, it involves systematic and coordinated practices through which an organization manages its assets, asset systems, performance, risks, and costs in a sustainable and optimized manner throughout their life cycles, with the aim of achieving strategic organizational objectives [10,11]. Although asset management practices vary across industries, implementing an Asset Management System (AMS) can play a crucial role in managing operations, security, sustainability, and efficiency for both tangible and intangible assets. Research and business analytics reports highlight the primary benefits of AMS, including cost reduction, increased asset utilization rates, risk management, improved decision-making, optimized maintenance strategies, and enhanced financial reporting [12,13]. Despite these advantages, the AMS often remains underrated in many organizations, leading to inefficiencies and operational challenges.

One sector that faces particular difficulties due to inadequate asset management is healthcare. Healthcare systems are among the most asset-intensive sectors globally, relying on advanced medical equipment, complex infrastructure, and digital platforms that require coordinated and proactive management. Hospitals and healthcare institutions are increasingly equipped with innovative medical technologies that improve diagnostics, treatment, and rehabilitation [14]. However, the absence of a well-integrated AMS results in several persistent challenges, such as inefficient tracking of medical equipment usage, inadequate maintenance planning, and regulatory non-compliance. These inefficiencies contribute to prolonged patient waiting times, financial losses, and reduced institutional credibility. Given that medical equipment is technologically complex, requires specialized expertise, and represents substantial financial investments, proper asset management is critical. Poorly managed medical assets can lead to underutilization, operational failures, and compromised patient care, ultimately affecting both financial sustainability and service quality [15]. Implementing an AMS in healthcare settings can help mitigate these risks by ensuring that assets are effectively utilized throughout their lifecycle, while meeting both patient and institutional needs. Yet, as our bibliometric analysis of 248 healthcare-specific AMS publications out of a total of 16,667 AMS documents shows, healthcare represents only a small fraction of the global AMS literature. This imbalance reveals a clear research gap between the strategic adoption of AMSs in industries such as engineering, energy or manufacturing and its comparatively fragmented implementation in healthcare. To address this gap, the present study systematically examines the thematic structure and evolution of AMS research both in general and within the healthcare domain, identifying emerging trends and highlighting areas of potential application in healthcare.

This study aims to analyze general research trends in the concept of asset management and assess the extent of its adoption in healthcare. The first phase provides a broad overview of asset management research, examining publication types, fields of study, and emerging topics. The second phase focuses on asset management in healthcare, identifying the most frequently researched areas, mapping study volume, content, publication trends, and research gaps. To achieve this, the study applies bibliometric analysis to documents on asset management, published up to the end of 2024 and indexed in the Scopus database. The research employs both established and innovative bibliometric techniques, leveraging Python software (version 3.13) to analyze publication trends, authorship patterns, and key thematic areas.

Following the introduction, Section 2 provides a review of existing research, offering insights into the concept of asset management and an overview of relevant bibliometric studies in the field. Section 3 outlines the methodological framework, detailing the bibliometric analysis approach and the features of the Python software utilized. Section 4 presents the research findings, mapping the landscape of AMS research, with a particular emphasis on its adoption in the healthcare sector. Section 5 engages in a critical discussion, addressing unresolved questions and evaluating the prospective benefits of AMS implementation in healthcare. Finally, Section 6 concludes with a summary of key findings and closing remarks on the potential role of asset management in the healthcare system.

## 2. Literature Review

Since the introduction of the asset management concept in the 1970s, when so-called ‘asset maintenance’ was initially regarded as a necessary burden, the field has undergone significant evolution. Today, it is recognized as a critical discipline for enhancing asset operations and value in a safe, efficient, and economical manner. Throughout this progression, asset management has evolved from a necessary operational function into a strategic and integral aspect of organizational decision-making. The discipline now involves optimizing the value of physical and financial assets through systematic processes that balance costs, risks, and performance. The application of asset management principles in healthcare is particularly crucial, given the need for patient safety, efficiency, sustainability, and regulatory compliance. This literature review section briefly synthesizes existing research and bibliometric studies on asset management, with a specific focus on its applications in healthcare.

Bibliometric analyses have mapped the evolution of asset management research, demonstrating increasing academic interest in recent decades. One of the most compelling reviews by Tajudin et al. analyzed over 4190 Scopus-indexed publications (between 1965 and 2020), revealing a steady increase in asset management research until the 1990s, and a significant rise in the 2000s [16]. The study indicates that, during the investigated period, the majority of studies were published in the form of journal articles and conference proceedings. In terms of content, the subject area is strongly dominated by engineering, followed by business, management and accounting, and computer science. According to this study, the linguistic distribution of studies on asset management research is uneven, with English accounting for more than 90% of publications. Similarly, geographical disparities exist, as most research originates from North America, Europe, China, Japan, Australia, and Canada, leaving significant gaps in developing regions.

Da Silva and de Souza conducted a bibliometric analysis of asset management research based on 2449 documents, confirming its rapid growth over the past 15 years, further driven by the international proliferation and adoption of the ISO 55000 standard [17]. In addition to standard bibliometric categories—such as publication trends, key research areas, main sources of publication, contributing authors, citation index, and international collaborations—their findings highlight a concentration of research in five key areas: finance and business, infrastructure, maintenance, optimization, and management. The conclusions regarding geographical disparities align with previous studies, as the same countries are identified as the most prolific publishers and key international collaborators in asset management research.

Another bibliometric study by Garramone et al. is more narrowly focused, specifically examining the integration of digital tools and systems in infrastructure asset management [18]. Based on the analysis of 54 journal articles, the authors highlight the role of specialized digital applications in establishing infrastructure asset management and the associated benefits and challenges. Similarly, other studies in the fields of infrastructure, construction, and related disciplines emphasize the importance of digital tools in asset management [19].

In the healthcare sector, although AMS research spans a wide range of thematic domains, it remains primarily concentrated on operational aspects—such as predictive maintenance of medical equipment and the implementation of digital systems for managing hospital facilities and infrastructure—rather than on the strategic integration of AMSs into clinical workflows and patient-centered care throughout the healthcare system. A bibliometric study conducted by Leiblein et al. analyzed abstracts of an initial selection of 177 journal articles to investigate facility management in hospitals, risk management of water systems, and associated infections [20]. In this context, their analysis primarily focused on the potential of asset risk management in hospital settings but did not explore broader trends in healthcare asset bibliometrics. Other studies, beyond bibliometric analyses, also confirm the increasing adoption of asset management in healthcare, recognizing its significant potential in integrating digital technologies with asset management concepts [21]. These studies frequently focus on the management of medical equipment, facilities, and digital resources to enhance administrative and operational efficiency. More recently, digital transformation—particularly through the implementation of AMSs incorporating technologies such as the IoT and AI—has emerged as a central research trend in healthcare asset management [22,23,24]. Despite these advancements, notable gaps remain in the current body of knowledge. While prior studies address topics such as facility maintenance, lifecycle optimization, and the integration of digital technologies, there is still a lack of comprehensive investigations into the broader impacts of enhanced asset management on patient care, healthcare service delivery, and treatment outcomes. In addition, analyses of AMS-related research, including bibliometric studies, typically employ methodologies such as co-word analysis, citation network mapping, and thematic clustering. For instance, bibliometric analyses in asset management frequently use topic modeling to identify key themes, including finance and business, management, infrastructure and risks, optimization, and maintenance, with a predominant focus on financial, managerial, and organizational aspects. However, these approaches often rely on terminology and conceptual frameworks originating from the corporate domain, which may not align with the specific contexts and language of healthcare. Consequently, many studies that substantively address asset management and its implications for healthcare tend to be overlooked, resulting in limited visibility and impact within both professional and academic communities.

Compared to the broader asset management field, healthcare-related AMS research remains underdeveloped, particularly in terms of systematic frameworks and patient-centered perspectives [25]. A key critique of the existing literature is its predominant focus on narrow, technology-driven dimensions of asset management, often at the expense of organizational, human, and resource-based considerations. Critical areas such as stakeholder engagement, employee participation, regulatory and decision-making structures, and integration with broader business processes are insufficiently addressed. Interdisciplinary perspectives—spanning public health, operations management, finance, and organizational studies—are also notably underrepresented, despite their clear relevance to healthcare asset management [7]. This research gap may be partially explained by the structural and operational characteristics of healthcare systems, which are typically rigid, heavily regulated, and resistant to rapid transformation. These factors present substantial barriers to the integration of innovative management concepts and emerging technologies. Within this context, effective implementation of AMSs in healthcare requires a paradigm shift encompassing comprehensive reforms in managerial practices, organizational structures, operational processes, regulatory frameworks, and information systems.

The evolution of asset management research has been relatively well-documented through bibliometric analyses, yet healthcare-specific studies remain fragmented and often overlooked. By employing more systematic research approach, including bibliometric methodologies, future studies can identify emerging trends, promote interdisciplinary collaboration, and provide evidence-based insights for policymaking and operational improvements in healthcare asset management. However, to comprehensively address asset management challenges in healthcare, research efforts must be expanded. Only in-depth research can contribute to the advancement of asset management in healthcare, ultimately leading to better resource utilization, enhanced patient care, and increased cost efficiency in healthcare systems worldwide.

## 3. Materials and Methods

### 3.1. Data Source and Rationale

Comprehensive bibliometric data on general AMS research were retrieved on 31 January 2025, while data specifically focusing on AMS research in the healthcare domain were systematically collected on 31 May 2025, both from Scopus, a leading international database of peer-reviewed literature. Scopus was selected after a careful evaluation of available bibliometric databases, for the following reasons. First, it has broader coverage of scientific research outputs compared to other widely used bibliometric databases such as Web of Science [26]. Second, Scopus provides superior coverage of the Social Sciences and Humanities, areas that are often underrepresented in Web of Science, but are highly relevant for AMS research given its interdisciplinary nature [27]. Third, Scopus was selected as the sole data source to ensure methodological consistency and reproducibility, as it provides broad interdisciplinary coverage across asset management, healthcare, engineering, and information systems. Fourth, although English remains the dominant language in both Scopus and Web of Science, Scopus offers greater inclusivity of non-English publications, while ensuring accessibility by providing English-language metadata in titles, abstracts, and keywords [28]. This ensures a globally representative sample while maintaining language consistency for analysis. Finally, in line with fundamental statistical principles, which are equally relevant in bibliometric analyses, larger sample sizes generally yield more robust and precise results, thereby facilitating more credible analytical outcomes [29]. Taken together, Scopus appears to be the more relevant bibliographic database, as it aligns well with the characteristics of AMS research.

### 3.2. Search Strategy and Data Collection

The search strategy was designed to ensure both comprehensiveness and replicability. An advanced search query was applied to the title, abstract, and keyword fields, targeting documents that explicitly mentioned “asset management” in any of these fields. Accordingly, the search query was structured as follows: TITLE-ABS-KEY ({asset management}), whereby the use of curly brackets denotes an exact search, retrieving only results where the words appear together in the precise order, in contrast to double quotation marks, which perform a loose phrase search often applied in medical research [30]. As the search query was intentionally broad to ensure the inclusion of the largest possible number of relevant documents and provide a comprehensive overview of AMS research, the use of an exact phrase search with curly brackets simultaneously reduced noise by limiting results to documents explicitly containing the term “asset management” in the specified fields. An identical search strategy was employed to perform a targeted bibliometric analysis of AMS research in the healthcare domain. The search focused on retrieving specific documents that included the phrase “asset management” in conjunction with at least one of the following healthcare-related terms: “healthcare,” “hospital,” “medical equipment,” or “medical devices.” This targeted strategy ensured that we captured only publications directly relevant to the intersection of AMSs and healthcare. Accordingly, the full search query was structured as follows: (TITLE-ABS-KEY ({asset management}) AND TITLE-ABS-KEY ({healthcare} OR {hospital} OR {medical equipment} OR {medical devices})).

No language restrictions were imposed in the search query. While the full texts of retrieved documents may be in any language, this does not affect the results, since Scopus indexes titles, abstracts, and keywords in English, thereby ensuring comparability and reliability in bibliometric processing. The time frame for inclusion was restricted to publications up to 31 December 2024, thereby avoiding distortions caused by partial data from 2025. This approach ensures year-on-year comparability and prevents disproportionate representation of data from incomplete publication years. The presented inclusion criteria were established to ensure the relevance, consistency, and methodological transparency of the dataset. Only peer-reviewed publications that addressed asset management as a substantive research focus and aligned with the ISO 55000 conceptual framework were retained for analysis. Studies concentrating exclusively on financial asset management or other non-organizational contexts were excluded to maintain conceptual coherence. Likewise, non-scientific materials such as grey literature and technical or institutional reports were omitted. These measures ensured that the final dataset comprised academically validated research directly pertinent to organizational and infrastructural asset management and its applications within the healthcare sector.

This search strategy returned a total of 16,667 documents related to AMS, forming the primary dataset for the subsequent bibliometric analysis, and 248 documents specifically addressing AMSs in healthcare, constituting a focused subset for further examination of the application of asset management in the healthcare sector. Metadata for each document included bibliographic details, citation information, countries of origin, publication sources, and keywords, forming the foundation for quantitative and network-based analyses.

### 3.3. Analytical Approach

To address the study objectives, a combination of quantitative bibliometric techniques and software tools was applied. The bibliometric analysis was performed using the Python data analysis libraries Pandas and NumPy [31], which facilitated data cleaning, normalization, and statistical processing. Duplicate records, if any, were removed, and document types (e.g., articles, reviews, conference papers) were classified. Standard bibliometric indicators were calculated, including annual publication trends, citation counts, countries of origin, publication sources (for general AMS research), and keywords. These indicators provided an overview of the evolution and structure of AMS research. Patterns and distributions were visualized using the Python visualization library Matplotlib [32], ensuring clarity in temporal trends and geographical outputs. To uncover intellectual and thematic structures, a network analysis, i.e., keyword co-occurrence, was conducted using VOSviewer (version 1.6.20), a widely recognized software tool for constructing and visualizing bibliometric networks [33]. This allowed for the identification of research clusters, thematic hotspots, and emerging topics in AMS research.

Beyond quantitative analysis, qualitative insights were incorporated to contextualize bibliometric findings. Abstracts of documents that explicitly discussed the role of AMSs in healthcare applications were screened to capture emerging perspectives, key challenges, and potential research gaps. This mixed-methods approach combined bibliometric rigor with targeted qualitative synthesis, offering a more comprehensive understanding of the field.

## 4. Results

**Figure 1:**
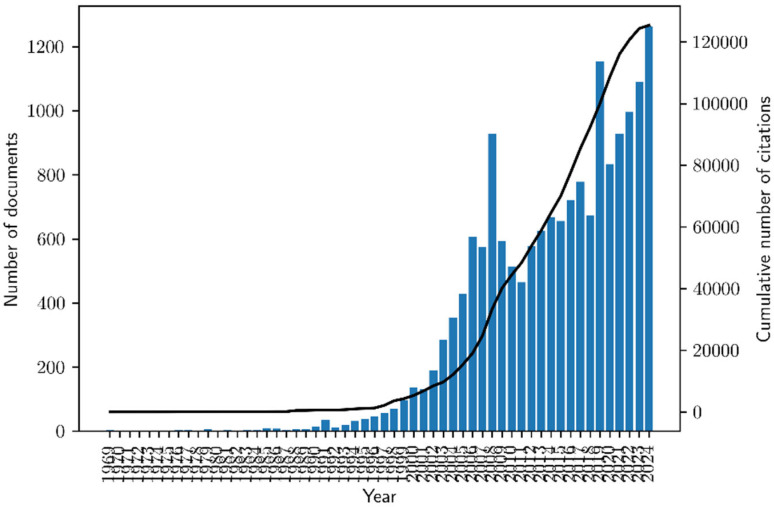
Scientific production in AMS research. Source: Own elaboration based on the Scopus database.

AMS research comprises 16,667 documents published in 5512 sources, including journals, books, and conference proceedings, between January 1969 and December 2024, accumulating a total of 125,396 citations. The majority of these documents are conference papers (48.6%) and articles (37.2%), followed by book chapters (4.5%), reviews (3.8%), conference reviews (2.1%), books (1.4%), and notes (1.0%). The remaining document types (1.3%) include short surveys, editorials, errata, retracted papers, data papers, letters, and reports. As shown in Figure 1, the scientific production in AMS research has grown substantially over the past decades, with a pronounced increase in publication output and citation impact. Before the 1990s, research activity was minimal due to the nascent stage of the AMS and its limited adoption across industries. From the mid-1990s onward, publications rose steadily, driven by advancements in digital asset management, predictive maintenance, and data-driven decision-making in asset-intensive sectors. This growth accelerated in the 2000s and 2010s, fueled by the widespread integration of ICTs, the rise of Industry 4.0, and the adoption of smart maintenance strategies enabled by the IoT and AI. Concurrently, increased focus on risk management, performance optimization, and infrastructure resilience reflected the transition toward proactive asset lifecycle management. The surge in publications from 2020 to 2024 highlights the growing impact of big data analytics, cloud computing, and AI-driven decision support systems, aligning with the need for more automated, predictive, and adaptive asset management solutions. This period also saw a marked rise in sustainability-focused AMS research, emphasizing regulatory compliance, circular economy principles, and long-term asset resilience in response to global economic and environmental challenges. These trends underscore the evolution of AMSs from traditional asset tracking to intelligent, data-centric, and sustainability-driven management frameworks.

**Figure 2:**
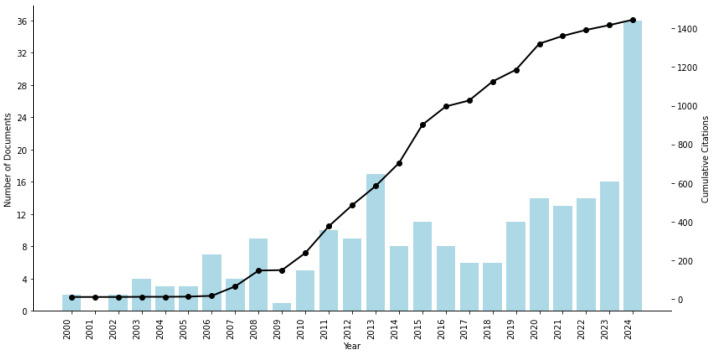
Scientific production in AMS research in healthcare. Source: Own elaboration based on the Scopus database.

Within this global landscape, healthcare-specific AMS research remains comparatively modest but is growing rapidly. Between 1979 and 2024, 248 healthcare-focused AMS documents were published. This subset accounts for approximately 1.49% of the global AMS literature indexed in Scopus. This figure should be interpreted as a rough estimate, since the end dates of the bibliometric analyses differed: bibliometric data on general AMS research were retrieved on 31 January 2025, whereas data on AMS research in healthcare were collected on 31 May 2025. The majority are journal articles (50.4%) and conference papers (31.9%), followed by book chapters (8.1%), conference reviews (3.2%), and review articles (2.8%). Books (1.6%), short surveys (1.2%), and notes (0.8%) represent smaller shares. Figure 2 depicts the trajectory of scientific production in AMS research within the healthcare domain between 2000 and 2024. Before 2000 (not displayed in Figure 2), AMS research in healthcare was limited, with just 28 documents published between 1979 and 1999. A noticeable increase began after the mid-2000s, reflecting growing academic interest in the role of asset management in health systems. From 2010 onward, both publication output and citation impact accelerated significantly, driven by technological adoption in healthcare and increased attention to operational efficiency, medical equipment optimization, and infrastructure resilience. This upward trend continued into the 2020s, culminating in a peak of scientific activity between 2020 and 2024, underscoring the increasing maturity and strategic relevance of AMSs in healthcare.

**Figure 3:**
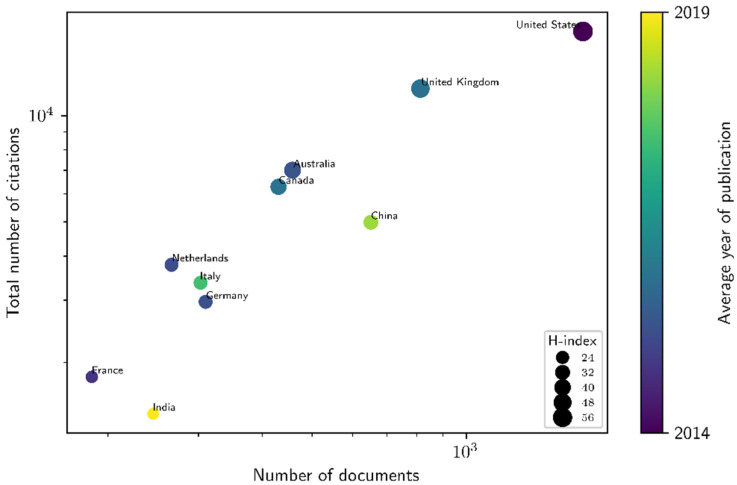
The most relevant countries in AMS research. Source: Own elaboration based on the Scopus database.

The most relevant countries in AMS research are presented in Figure 3, highlighting the most influential ones based on the number of documents (*x*-axis), total citations (*y*-axis), H-index (circle size), and average publication year (circle colour). The global landscape of AMS research is led by the United States, which stands out with the highest number of published documents (1687) and citations (17,309), along with the highest H-index (59), reflecting its significant impact and influence in the field. The United Kingdom maintains a strong research presence with the second-largest number of documents (813) and citations (11,927), supported by a relatively high H-index (51) and a more recent average publication year compared to the United States. Meanwhile, China has emerged as a rapidly growing contributor, ranking third in both documents (651) and citations (4987), with one of the most recent average publication years, signaling a research landscape that is expanding but still maturing. Similarly, Australia and Canada (with 458 and 430 documents, respectively) demonstrate moderate citation impact, indicating an active yet more regionally concentrated research community. In contrast, Germany, the Netherlands, and Italy contribute significantly but with fewer total citations, suggesting a more specialized research focus within AMS. While France and India report lower research outputs, India’s publications are among the most recent, reflecting a rising interest in the field. Overall, the trend indicates that countries with a longer history of AMS research, such as the United States and the United Kingdom, maintain higher citation impact, whereas newer contributors like China and India are rapidly increasing their research activity. The growing presence of countries with recent publication trends highlights an expanding global interest in AMS, driven by technological advancements, data-driven methodologies, and the integration of AI-powered asset management solutions.

**Figure 4:**
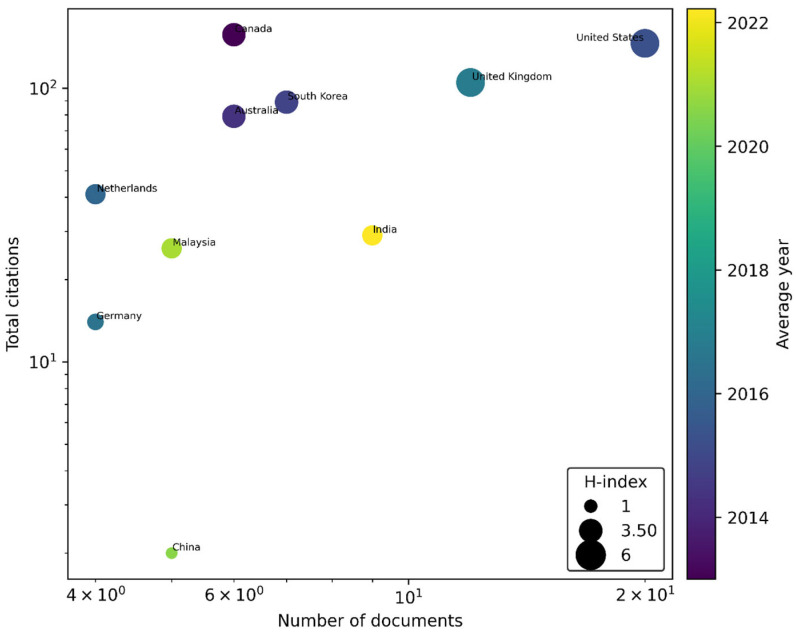
The most relevant countries in AMS research in healthcare. Source: Own elaboration based on the Scopus database.

Figure 4 provides a comparative illustration of the leading countries contributing to AMS research in healthcare. The visualization is based on the number of published documents and total citations, both represented on logarithmic scale. Bubble size corresponds to the H-index, capturing the combined effect of research productivity and citation impact, while the color gradient reflects the average year of publication, with lighter shades indicating more recent activity. The results reveal that the United States and the United Kingdom dominate in both research output and citation impact, underscoring their long-standing and active engagement in AMS research. Countries such as Canada, South Korea, and Australia also demonstrate significant influence, achieving relatively high citation counts despite lower publication volumes. In contrast, India, Malaysia, and China emerge as newer contributors, characterized by increasing research output and a growing citation footprint. Taken together, the figure illustrates a global research landscape shaped by both well-established leaders and rapidly emerging contributors in AMS research within the healthcare sector.

**Figure 5:**
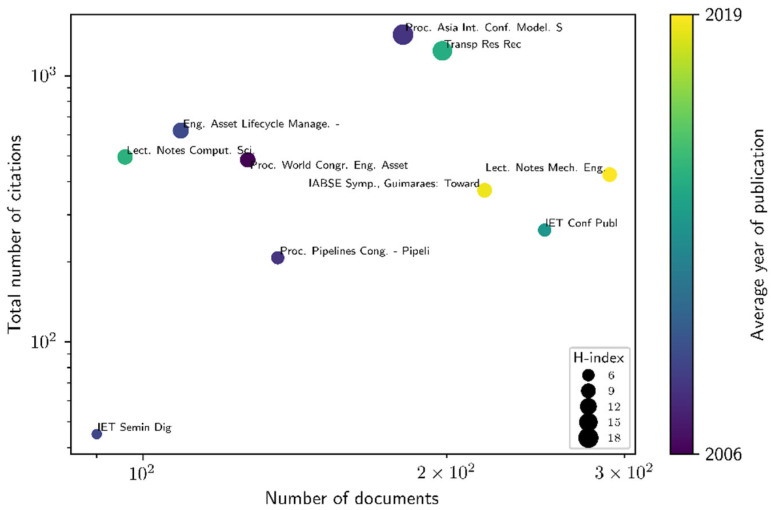
The most relevant sources in AMS research. Source: Own elaboration based on the Scopus database.

The most relevant sources in AMS research are presented in Figure 5, highlighting the most influential ones based on the number of documents (*x*-axis), total citations (*y*-axis), H-index (circle size), and average publication year (circle colour). Among them, *Lecture Notes in Mechanical Engineering* stands out with the highest number of documents (290) and citations (425), positioning it as the most significant contributor to AMS research. Following closely, *IET Conference Publications* holds the second-largest number of documents (250) and citations (263), demonstrating strong research output but with a slightly lower citation impact. In a similar vein, the *IABSE Symposium* ranks third with 218 documents and 371 citations, reflecting a notable research presence with moderate influence. Additionally, *Transportation Research Record* and *Proceedings of the 2nd Asia International Conference on Modeling & Simulation* (with 198 and 181 documents, respectively) contribute significantly, highlighting their role in AMS-related discussions. On the other hand, sources such as *Engineering Asset Lifecycle Management, World Congress on Engineering Asset Management*, and *Lecture Notes in Computer Science* have fewer total citations, suggesting a more specialized research focus within AMS. Meanwhile, *JET Seminar Digest* and *Proceedings of the Pipelines Conference* report lower research outputs, indicating a niche but focused interest in specific AMS topics. Overall, the trend suggests that sources with a longer history of AMS publications maintain higher citation impact, whereas newer sources are gaining recognition through more recent contributions. The increasing number of documents in recent years highlights a growing research interest in AMS, reflecting advancements in asset management strategies, digitalization, and sustainability-driven methodologies.

In light of the limited number of documents identified by the bibliometric analysis as covering AMS research in healthcare, and considering the relatively recent rise in scholarly interest in this area (with a significant growth in both publication output and citation impact only since 2010), the scattered distribution of sources and the absence of well-established journals or specific outlets addressing this topic were not unexpected. Accordingly, a focused analysis of the most relevant sources in AMS research in healthcare did not produce sufficiently informative results and has therefore been omitted from this article.

**Figure 6:**
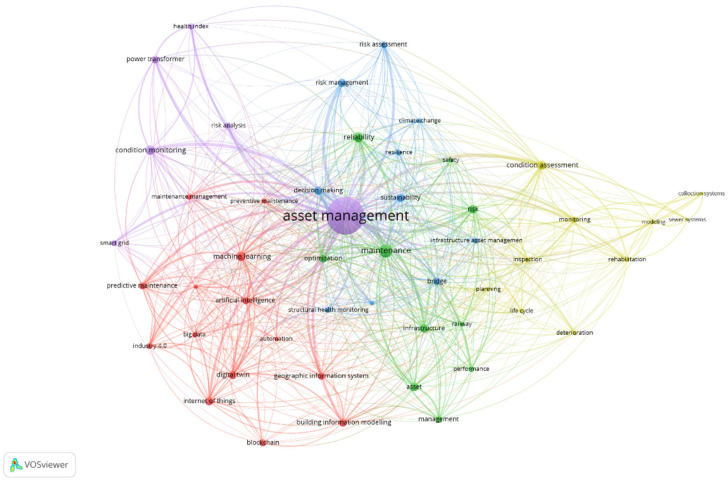
Keyword co-occurrence network of AMS research. Source: Own elaboration based on the Scopus database.

**Figure 7:**
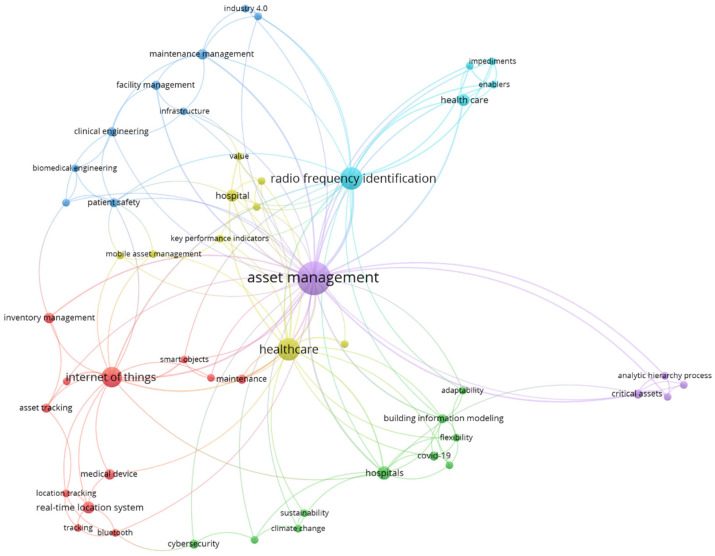
Keyword co-occurrence network of AMS research in healthcare. Source: Own elaboration based on the Scopus database.

The network analyses of AMS research and AMS research in healthcare, i.e., keyword co-occurrences, performed on the 50 most frequent authors keywords, are presented in Figure 6 and Figure 7. Note that the nodes represent keywords, while the links indicate co-occurrence relationships between them. The node size is proportional to the number of keyword occurrences, showing research intensity (node degree), while the link width is proportional to the co-occurrences between keywords (edge weight). Additionally, the node color indicates the cluster to which a particular keyword belongs [34]. The analysis identifies five key hotspots in general AMS research, each addressing a prominent area of interest (Figure 6): (1) emerging technologies in maintenance and infrastructure (red cluster), (2) core infrastructure and reliability management (green cluster), (3) risk and resilience in infrastructure (blue cluster), (4) infrastructure condition assessment and rehabilitation (yellow cluster), and (5) smart grid and asset monitoring (purple cluster). However, the network analysis of AMS research in healthcare revealed a distinct set of six hotspots, reflecting the specific nature of the healthcare sector, its operational characteristics, and management practices (Figure 7): (1) smart technologies and asset tracking (red cluster), (2) risk and sustainability (green cluster), (3) engineering and operational systems (dark blue cluster), (4) performance value management in healthcare logistics (yellow cluster), (5) strategic asset capital planning (purple cluster), and (6) patient centric enablers and barriers (light blue cluster).

A detailed synopsis of the research hotspots for AMS research in general and AMS research in healthcare, including the representative (most frequent) author keywords, is presented in Table 1 and Table 2.

The first cluster (Table 1), emerging technologies in maintenance and infrastructure, explores the integration of artificial intelligence, automation, and digital transformation in optimizing infrastructure management. These advancements enable predictive and preventive maintenance, reducing costs and improving efficiency [7]. Technologies such as digital twins, IoT, and big data analytics are reshaping the way infrastructure is monitored, ensuring real-time insights and proactive decision-making.

The second cluster (Table 1), core infrastructure and reliability management, focuses on ensuring the safety, reliability, and optimization of critical infrastructure systems. Research in this domain emphasizes performance monitoring, maintenance strategies, and risk assessment to minimize operational failures [18]. As infrastructure networks expand, managing their long-term functionality and efficiency becomes increasingly vital. This cluster underscores the role of structured asset management frameworks in sustaining essential infrastructure such as railways, bridges, and transport systems.

The third cluster (Table 1), risk and resilience in infrastructure, delves into the challenges posed by climate change, structural deterioration, and decision-making complexities in infrastructure management. With growing environmental uncertainties and extreme weather events, resilience strategies have become crucial in safeguarding infrastructure assets [5]. Research in this area highlights risk assessment, resilience frameworks, and structural health monitoring, ensuring that infrastructure systems remain functional and sustainable under adverse conditions.

The fourth cluster (Table 1), infrastructure condition assessment and rehabilitation, addresses the need for systematic evaluation and restoration of aging infrastructure. This research hotspot focuses on deterioration modeling, life cycle assessment, and monitoring techniques to detect structural weaknesses [18]. Effective rehabilitation strategies help extend the lifespan of critical assets, improving their serviceability while reducing maintenance costs. By leveraging data-driven assessment methods, this cluster aims to optimize infrastructure rehabilitation and long-term performance.

The fifth cluster (Table 1), smart grid and asset monitoring, examines the application of smart technologies in energy distribution and infrastructure management. With the increasing adoption of smart grids, asset monitoring has become essential for ensuring energy efficiency, grid stability, and risk mitigation. Condition monitoring techniques and advanced analytics play a key role in assessing the health of power systems, transformers, and other critical assets [15]. This cluster highlights the transformative potential of digital solutions in optimizing infrastructure performance and enhancing sustainability.

The first cluster (Table 2), smart technologies and asset tracking, highlights the integration of advanced digital tools, automation, and interconnected systems that enable real-time monitoring, location tracking, and more efficient asset utilization in healthcare. This line of research underscores how IoT and wireless communication can enhance operational transparency, minimize asset loss, advance infrastructure maintenance, and support data-driven decision-making. AI-driven prognostics and smart hospital facility management systems are increasingly used to improve medical equipment utilization, break down data silos, and optimize hospital operations, improving coordination and patient care [35].

The second cluster (Table 2), risk and sustainability, reflects a growing recognition of AMS as a tool for building resilient and environmentally responsible healthcare systems [36]. It underscores how asset management supports preparedness for external shocks—such as pandemics and climate change—while promoting sustainable infrastructure practices [37]. Designing climate-resilient hospitals is an emerging priority, as healthcare facilities must withstand environmental risks while maintaining critical services. Sustainable hospital planning now incorporates asset management strategies to enhance energy efficiency and structural resilience [38]. Risk and sustainability management are critical for ensuring the continuous operation of medical devices and hospital facilities. Studies show that hospitals suffering from deferred maintenance in their physical infrastructure can experience disruptions in the provision of health services and financial losses, highlighting the importance of proactive asset management in mitigating risks and sustaining operational efficiency [39].

The third cluster (Table 2), engineering and operational systems, underscores the foundational role of clinical and biomedical engineering and the digital transformation strategies that enable efficient facility management, system integration, and continuous operational improvement. This cluster also reflects the evolution of AMS in line with Industry 4.0 and 5.0, pointing toward intelligent, adaptive, and interconnected healthcare environments. The adoption of Enterprise Building Information Modeling (BIM) is revolutionizing hospital asset management. Research demonstrates how Norwegian hospitals are using BIM for real-time monitoring of physical assets, improving maintenance planning, operational efficiency, and reducing downtime for critical medical facilities [40].

The fourth cluster (Table 2), performance value management in healthcare logistics, focuses on enhancing the value generated by AMS through key performance indicators, real-time data use, and supply chain optimization. Research in this area seeks to improve the efficiency, reliability, and responsiveness of logistics operations to ultimately strengthen patient outcomes and organizational performance. Different studies on hospital asset tracking have shown that digital transformation is key to ensuring seamless operations and improving clinical workflows, contributing to more effective patient care [41].

The fifth cluster (Table 2), strategic asset capital planning, emphasizes long-term investment strategies, lifecycle analysis, and prioritization frameworks for managing healthcare infrastructure and medical devices. It highlights AMS’s role in supporting evidence-based decisions regarding capital renewals, critical assets, and infrastructure upgrades—particularly under conditions of financial constraint and rising demand. Finally, the sixth cluster (Table 2), patient-centric enablers and barriers, addresses the human, organizational, and technological factors shaping the adoption and impact of AMS initiatives. This research direction stresses the importance of aligning asset management with patient care objectives, addressing user needs, overcoming implementation challenges, and ensuring that technology enhances rather than hinders the patient experience.

## 5. Discussion

### 5.1. Evolution and Thematic Trends in AMS Research

The findings of this bibliometric analyses highlight the rapid evolution and increasing significance of AMS in various fields, with a particular emphasis on its role in healthcare. The substantial growth in scientific production over the past decades demonstrates that AMS has transformed from a niche research area into a strategic discipline that enhances infrastructure resilience, operational efficiency, and sustainability [42]. Our analysis confirms key trends identified in previous research, reinforcing the notion that AMS is progressively being integrated into multiple sectors, including healthcare [43], where its impact on hospital infrastructure, medical equipment management, and associated costs, and risk management is becoming more pronounced.

The bibliometric trends identified in our studies indicate that AMS research has experienced substantial growth, particularly since the mid-1990s, due to advancements in digitalization, predictive analytics, and asset-intensive industry applications. The expansion of AMS research in healthcare gained further momentum after the 2010s, driven by the widespread integration of ICTs and the rise of Industry 4.0. The period between 2020 and 2024 marks a significant acceleration in AMS research, largely influenced by big data analytics, AI, and cloud computing. The growing emphasis on sustainability-focused AMS research reflects the increasing need for regulatory compliance, circular economy principles, and resilience against economic and environmental challenges. Furthermore, the analysis of research hotspots through keyword co-occurrence networks has allowed for a more detailed classification of key themes in general AMS research and AMS research in healthcare, demonstrating an array of notable differences in focus, structure, and research trends over the past years.

Our study aligns with existing literature regarding the expansion and strategic importance of AMS. Previous research has consistently documented the increasing adoption of AMS in diverse fields, highlighting its role in enhancing efficiency, decision-making, and lifecycle management [44]. The distribution of AMS publications by type, source, and country generally follows patterns observed in prior studies. The dominance of conference papers and journal articles corroborates the notion that AMS research remains a dynamic and evolving field with strong industry and academic engagement [45]. Geographically, the United States and the United Kingdom continue to lead AMS research, as seen in past studies. However, our findings reveal the increasing contributions from China and India, indicating a shift in the global research landscape. In terms of research focus, this study builds on previous bibliometric analyses by offering a more detailed classification of AMS research hotspots both in general and within the healthcare domain. While previous studies have recognized broad themes such as maintenance management and infrastructure optimization [46], our research systematically defines the representative keywords within each research cluster in both fields. This enhanced granularity provides a more precise understanding of AMS research evolution and its alignment with emerging technologies and sustainability initiatives.

The research on asset management in healthcare has been steadily increasing in recent years. The bibliometric analysis of AMS in healthcare specifically reveals a growing research focus on predictive maintenance of medical equipment, hospital infrastructure optimization, and data-driven asset lifecycle management [47]. The rising adoption of AI-driven diagnostics, digital twin models, and IoT-enabled hospital asset tracking has significantly contributed to AMS research in healthcare. The literature suggests that AMS in healthcare plays an important role in minimizing operational disruptions and ensuring cost-effective maintenance strategies [48]. Furthermore, bibliometric trends indicate that the integration of sustainability and risk management in healthcare AMS research has gained traction [43], emphasizing energy-efficient hospital designs, resilient healthcare facilities, and strategic resource allocation.

### 5.2. Health Economics, Policy, and Analytical Implications of the Research Findings

The introduction of AMS in the healthcare context is not merely a technological undertaking; it also constitutes a significant economic intervention, with important implications for health service delivery, cost-effectiveness, and the fiscal sustainability of healthcare systems [49]. From a health economics perspective, the deployment of AMS and the accompanying digital transformation in healthcare has the potential to improve allocative efficiency, optimize resource utilization, and provide a foundation for the long-term rationalization of health expenditure [50]. The effective application of AMS can optimize the use of scarce healthcare resources by streamlining clinical workflows, minimizing medical errors, and reducing administrative redundancies, ultimately generating tangible operational efficiencies [51].

Digital tools embedded within AMS can improve scheduling, strengthen patient treatment adherence, and reduce the unnecessary duplication of diagnostics, thereby directly contributing to cost savings and efficiency gains [52]. For example, digital appointment systems integrated with AMS can lower waiting times and better align the supply and demand of healthcare services, which enhances both allocative efficiency and public health outcomes.

Furthermore, AMS enables real-time asset tracking, predictive analytics, and transparent reporting. These functions support more effective workforce deployment, capacity planning, and evidence-based policy formulation [53]. Although the initial investment in AMS and related digital infrastructure can be capital-intensive, such investments typically yield a high return over time by lowering operational costs, reducing administrative burdens, and improving preventive care, thereby relieving long-term fiscal pressures on healthcare budgets [54]. As a result, the introduction of AMS should be regarded as a strategic investment in healthcare productivity and a catalyst for reform. In the context of growing healthcare demand—driven by demographic shifts and the rising prevalence of chronic diseases—digitally assisted healthcare operations can contribute to better population health, greater system resilience, and economic sustainability, thereby supporting broader societal objectives such as economic growth, fiscal consolidation, and more efficient healthcare delivery [55]. The findings of this study suggest that the successful introduction of AMS in healthcare requires moving beyond technological perspective to an approach that integrates economic and governance dimensions. This reinforces the understanding of digitalization as a form of public capital investment, generating multifaceted returns in terms of financial performance, institutional resilience, transparency, and long-term adaptability of healthcare systems.

From a policy design standpoint, the study highlights the importance of aligning digital innovations, such as AMS implementation, with broader healthcare reforms [56]. Effective digital transformation requires inter-departmental coordination, harmonized regulatory frameworks, and inclusive stakeholder engagement. Such policy coherence enhances the adaptive capacity of healthcare institutions and ensures that digital reforms translate into measurable improvements in both service delivery and fiscal performance. Accordingly, this study positions AMS implementation as an integral component of comprehensive healthcare sector reform. Ultimately, the success of AMS adoption depends on its ability to integrate technological innovations with clinical aspects and economic considerations. Balancing emerging technologies with improved patient outcomes and cost-efficiency must remain central to AMS implementation strategies.

For the sake of the study’s objectivity, it is important to emphasize that, within the healthcare context, the nature of assets fundamentally differs from that in infrastructure or industrial systems. Traditional AMS frameworks are typically designed around passive, physical assets, whereas in healthcare, the core assets are human capital (clinical staff) and service delivery processes. Medical equipment, although highly important, generally plays a supporting or enabling role, rather than representing the primary productive asset. A key concept here is asset specificity: the economic value and operational utility of healthcare assets are context-dependent, often tied to particular service lines, locations, and staff expertise. This differs from generic physical assets, which are typically substitutable and transferable across settings with minimal loss of value. Accordingly, AMS frameworks cannot be transplanted into healthcare by analogy alone. Instead, they require structural adaptation that accounts for the high specificity and criticality of healthcare assets, as well as their interdependence with clinical workflows and regulatory regimes. This implies shifting from asset-centric models to service-centric asset management, where the optimization target is not merely asset uptime but continuity and quality of patient care. Moreover, institutional rigidity and regulation are not merely background constraints but central design and implementation parameters. These factors shape how AMS principles can be operationalized without creating excessive organizational disruptions. For example, predictive maintenance approaches must be balanced against clinical scheduling priorities, and asset tracking must be aligned with data governance and information security protocols. AMS projects involve profound transformations in routines, behaviors, work practices, organizational processes, and the overall management of healthcare. As such, they call for incremental and adaptive approaches rather than disruptive changes, in recognition of the complex and sensitive nature of healthcare systems. By reframing AMS not as a direct technological solution but as an adaptive managerial and organizational framework, all implementation efforts should better address the unique function of healthcare systems, where service provision depends on coordinated interaction between human, technological, and institutional assets.

In summary, although both foundational and domain-specific research on AMS in healthcare exists, a holistic and systematic understanding, as well as studies focusing on direct patient-centric outcomes, are still lacking. While existing studies indirectly address key healthcare concerns [23], such as patient safety and treatment quality, coordination of healthcare providers, reduction in patient waiting times, standardization of business processes, minimization of medical equipment downtime, and overall regulatory compliance, these areas require more in-depth investigation. Effective asset management has the potential to enhance operational efficiency, improve healthcare outcomes, and strengthen public trust in healthcare institutions [48], yet its role in these domains remains insufficiently examined. Additionally, the influence of asset management on strategic decision-making within the healthcare sector is an area that has received minimal research attention. Few studies explore how asset management contributes to financial planning, organizational strategy, human resource allocation, joint procurement strategies, productivity control, and the identification of gridlocks in healthcare program implementation. Furthermore, its role in shaping public health policies and operative measures remains largely disregarded.

The implications of our findings are manifold. First, they highlight the necessity of continued investment in AMS research to address asset management challenges, particularly in healthcare, where asset reliability and operational efficiency are critical. The identification of emerging technologies such as AI, IoT, and digital twins within AMS research underscores the growing demand for intelligent, automated asset management solutions. The shift toward sustainability-oriented research reflects the increasing pressure on organizations to comply with operational requirements, environmental regulations and implement long-term resilience strategies. Policymakers, industry leaders, and researchers can leverage these insights to drive innovations in AMS, fostering a more comprehensive, proactive and data-driven approach to asset management.

### 5.3. Limitations of the Study and Future Research Orientations

Despite its comprehensive scope, this study has certain limitations. The analysis relies solely on bibliometric data from the Scopus database. Although Scopus provides broad coverage, the exclusion of other databases such as Web of Science or IEEE Xplore may have introduced selection bias and limited the comprehensiveness of the datasets. In addition, the keyword-based search strategy is relatively general and may have excluded relevant studies, particularly in fields such as healthcare, where different terminology is often used. Moreover, the reliance on keyword co-occurrence analysis means that emerging but less frequently used terms might not be fully represented in the research. Finally, while the study provides a global overview of AMS research, it does not account for the specific methodologies employed in individual studies, limiting the ability to assess the methodological rigour of the field.

Future research should explore the methodological approaches used in AMS studies to assess their effectiveness and impact. Expanding the analysis to include other bibliometric databases could provide a more comprehensive view of the global AMS research landscape. Additionally, longitudinal studies examining the practical implementation of AMS solutions in various industries, particularly healthcare, could offer valuable insights into the real-world benefits, policy impacts, and challenges of asset management strategies. As AI and IoT continue to reshape AMS, further investigation into their integration and effectiveness in asset management frameworks will be essential. Given the identified gaps, a comprehensive research framework for healthcare asset management should integrate elements from patient care domain, medical equipment and facility management, cost efficiency, and technology adoption theories. Moreover, interdisciplinary research that connects healthcare AMS with public health issues, health economics and management, modern technologies, and sustainability will be crucial for ensuring that AMS evolves in alignment with emerging health, societal, and technological needs.

## 6. Conclusions

This study highlights the rapid growth and increasing importance of AMSs across various industries and applications. Over the past few decades, the AMS has evolved from a niche research topic into a strategic discipline that enhances operational efficiency, predictive maintenance, and sustainability. The findings confirm key trends observed in previous research, demonstrating that the AMS is progressively integrating into multiple sectors, including healthcare, where it significantly impacts medical equipment management, hospital infrastructure reliability, and risk mitigation. The bibliometric analysis identifies substantial growth in AMS research since the mid-1990s, largely driven by advancements in digitalization and asset-intensive industry applications. This trend accelerated after the 2010s, especially in healthcare, due to the widespread adoption of ICTs and Industry 4.0, with an even more pronounced increase after 2020, fueled by big data analytics and AI. Additionally, sustainability-focused AMS research has gained prominence, reflecting the growing need for regulatory compliance, circular economy principles, and resilience against economic and environmental challenges.

Despite the different context and role of the asset management concept in healthcare, AMS research has expanded substantially in recent years, particularly in the areas of predictive maintenance of medical equipment, hospital infrastructure optimization, and data-driven asset lifecycle management. The adoption of emerging technologies, including AI and IoT-enabled asset tracking, has played a crucial role in improving healthcare asset management. However, a key gap in the literature is the limited focus on the AMS’s direct impact on healthcare delivery, including treatment quality, patient safety, healthcare provider coordination, and the reduction in patient waiting times. Furthermore, the AMS’s role in strategic decision-making, financial planning, and healthcare policy development remains underexplored.

The findings underscore the need for continued investment in interdisciplinary AMS research, particularly in healthcare, where asset reliability and operational efficiency are critical. The increasing integration of emerging technologies in AMSs highlights the growing demand for intelligent, automated asset management solutions. Promoting the strategic role and potential applications of the AMS in underrepresented sectors such as healthcare requires further exploration of its tangible benefits for patient care and its financial advantages for the broader healthcare system. This is particularly relevant in the context of current public health challenges and financial constraints amid increasingly uncertain global economic conditions.

## Figures and Tables

**Table 1:** Research hotspots based on the keyword co-occurrence network of AMS research.

Research Hotspots	Representative Keywords
Emerging technologies in maintenance and infrastructure	artificial intelligence, automation, big data, blockchain, building information model, digital twin, digitalization, geographic information, Industry 4.0, internet of things, machine learning, maintenance management, predictive maintenance, preventive maintenance
Core infrastructure and reliability management	asset, infrastructure, maintenance, management, optimization, performance, railway, reliability, risk, safety
Risk and resilience in infrastructure	bridge, climate change, decision making, decision support, infrastructure asset management, resilience, risk assessment, risk management, structural health monitoring, sustainability
Infrastructure condition assessment and rehabilitation	collection systems, condition assessment, deterioration, inspection, life cycle, modeling, monitoring, planning, rehabilitation, sewer systems
Smart grid and asset monitoring	asset management, condition monitoring, health index, power transformer, risk analysis, smart grid

Source: Own elaboration based on the Scopus database.

**Table 2:** Research hotspots based on the keyword co-occurrence network of AMS research in healthcare.

Research Hotspots	Representative Keywords
Smart technologies and asset tracking	5 g, asset tracking, Bluetooth, internet of things, inventory management, location tracking, logistics, maintenance, medical device, real-time location system, smart objects, tracking
Risk and sustainability	adaptability, building information modeling, climate change, COVID-19, cybersecurity, flexibility, hospitals, pandemic, risk management, sustainability
Engineering and operational systems	biomedical engineering, clinical engineering, enterprise resource planning, facility management, Industry 4.0, Industry 5.0, infrastructure, maintenance management, patient safety
Performance value management in healthcare logistics	critical infrastructure, healthcare, hospital, key performance indicators, mobile asset management, performance, real-time location system, supply chain management, value
Strategic asset capital planning	analytic hierarchy process, asset management, capital renewals, critical assets, healthcare facilities
Patient centric enablers and barriers	enablers, healthcare, impediments, patient management, radio frequency identification

Source: Own elaboration based on the Scopus database.

## Data Availability

The original data presented in the study are openly available in the Scopus database and can be obtained from the corresponding author upon reasonable request.
